# ^11^C-PiB PET/MRI在原发性系统性轻链型淀粉样变器官受累评估中的价值

**DOI:** 10.3760/cma.j.issn.0253-2727.2022.04.009

**Published:** 2022-04

**Authors:** 雅丹 王, 雅景 杨, 盈盈 武, 春艳 孙

**Affiliations:** 华中科技大学同济医学院附属协和医院血液病研究所，武汉 430022 Institute of Hematology, Union Hospital, Tongji Medical College, Huazhong University of Science and Technology, Wuhan 430022, China

**Keywords:** 淀粉样蛋白, 正电子发射断层显像术, 系统性轻链型淀粉样变, 器官, Amyloid, Positron-emission tomography, Systemic light chain amyloidosis, Organ

## Abstract

**目的:**

分析^11^C标记的匹兹堡化合物B（^11^C-PiB）PET/MRI在原发性系统性轻链型淀粉样变（pAL）中评估器官受累的价值。

**方法:**

回顾性分析2019年1月至2021年10月在华中科技大学同济医学院附属协和医院就诊的20例pAL患者及3名健康志愿者的临床资料，比较临床标准评估器官受累和PET/MRI评估的相关性，分析心脏相关生物学指标、疾病分期与心脏最大标准摄取值（SUVmax）之间的关系及24 h尿蛋白定量与肾脏SUVmax之间的关系。

**结果:**

①纳入20例患者，初诊患者18例，非初诊患者2例。观察到^11^C-PiB摄取阳性的脏器分别为：心脏15例（75％），肺部8例（40％），骨髓10例（50％），肌肉10例（50％），舌肌7例（35％），甲状腺6例（30％），唾液腺4例（20％），脾脏2例（10％），胃壁1例（5％）。②^11^C-PiB在心脏及骨髓摄取的阳性率与临床评估标准具有很好的相关性。在肺组织、脾脏、腺体、肌肉和舌肌中，^11^C-PiB PET/MRI评估的阳性率明显高于临床标准。但对于神经系统受累和脂肪组织的评估^11^C-PiB PET/MRI具有局限性。③分析18例初诊患者心脏相关生物学指标与心脏SUVmax之间的关系。左心室射血分数（LVEF）<50％且室间隔厚度（ISV）≥1.2 cm的患者相较LVEF≥50％且ISV<1.2 cm的患者其心脏SUVmax更高（*P*<0.05）。心脏SUVmax值在Mayo2004分期、Mayo2012分期各期之间差异有统计学意义，分期越晚，SUVmax值越高（*P*<0.05）。心脏SUVmax与心肌肌钙蛋白酶Ⅰ、N 末端前体脑钠肽水平呈明显正相关（*P*<0.01），肾脏SUVmax与24 h尿蛋白定量无明显相关性（*P*>0.05）。

**结论:**

全身^11^C-PiB PET/MRI作为一种淀粉样蛋白的可视化系统，若用于器官水平的定性评估，具有提高早期无创性诊断pAL水平的潜力；用于器官水平（尤其是心脏）的定量评估，有望更精准评估器官功能和预测疾病预后。

## Abstract (en)

**Objective:**

To analyze the value of ^11^C-PiB PET/MRI for evaluating organ involvement in patients with primary light chain amyloidosis（pAL）.

**Methods:**

The clinical data of 20 patients with pAL and 3 healthy volunteers from January 2019 to October 2021 were retrospectively analyzed. The correlation between the organ involvement evaluated by clinical standards and PET/MRI was compared. The relationship between cardiac-related biological indicators, disease stage, and the maximum standardized uptake value（SUVmax）were analyzed. The relationship between 24-hour urinary protein quantification and kidney SUVmax was analyzed.

**Results:**

①In 20 patients (18 newly diagnosed patients and 2 non-newly diagnosed patients), ^11^C-PiB positive uptake was observed in the heart（15 patients, 75％）, lung（8 patients, 40％）, bone marrow（10 patients, 50％）, muscle（10 patients, 50％）, tongue muscle（7 patients, 35％）, thyroid（6 patients, 30％）, salivary gland（4 patients, 20％）, spleen（2 patients, 10％）, and stomach wall（1 patient, 5％）. ②Organ involvement on ^11^C-PiB PET/MRI showed good correlations with the clinical evaluation criteria for the heart and bone marrow. The positive rate of PET/MRI evaluation in the lung, spleen, gland, muscle, and tongue muscle was significantly higher than the clinical criteria. However, ^11^C-PiB PET/MRI has limitations in the evaluation of the nervous system and fat tissue. ③To analyze the relationship between cardiac-related biological indexes and the SUVmax of the heart in 13 newly diagnosed patients. Patients with left ventricular ejection fraction（LVEF）<50％ and interventricular septal thickness（ISV）≥1.2 cm showed a higher SUVmax than patients with LVEF ≥50％ and ISV<1.2 cm（*P*<0.05）.There are significant differences in the SUVmax of the heart between the Mayo2004 stage and the Mayo2012 stage. The later the disease stage, the higher the SUVmax（*P*<0.05）. The SUVmax of the heart was positively correlated with cardiac troponin I（cTnI）and N-terminal pro-brain natriuretic peptide（NT-proBNP）（*P*<0.01）.There was no significant correlation between renal SUVmax and 24-hour urine protein（*P*>0.05）.

**Conclusion:**

Whole body ^11^C-PiB PET/MRI, as a visualization system of amyloid protein, is used to qualitatively evaluate organ involvement, which can improve the level of early non-invasive diagnosis. Whole body ^11^C-PiB PET/MRI can be used to perform quantitative evaluation of organ levels, especially the heart, which is expected to evaluate organ function and predict disease prognosis more accurately.

原发性系统性轻链型淀粉样变（primary light chain amyloidosis，pAL）是系统性淀粉样变中最常见的一种类型，是由浆细胞异常增殖产生的单克隆免疫球蛋白轻链错误折叠，形成淀粉样蛋白沉积至多个组织器官，从而引起器官功能障碍的一种疾病[1]。pAL临床表现多样且大多缺乏特异性，易被误诊和漏诊。pAL患者预后差，Mayo2004Ⅲ期患者中位生存期仅有3.5个月，其中心脏受累是患者死亡的主要原因[2]。因此早期诊断、早期识别器官受累至关重要。目前对于pAL的诊断，仍旧依赖于对组织进行活检，以确认轻链型淀粉样蛋白沉积。然而，这个方法具有明显的局限性，如相对较高的创伤性和对特定操作人员较高的技术要求。此外，组织活检只能用于评估单个器官的有限区域的淀粉样蛋白沉积。因此，全身淀粉样蛋白成像成为一种新兴的、无创的、可视化评估系统性淀粉样变的方法。^11^C标记的匹兹堡化合物B［^11^C-PiB，2-（4′-甲基苯基）-6-羟基苯甲噻唑］正电子发射断层显像术（PET）是一种成熟的淀粉样蛋白成像技术，PiB是淀粉样蛋白结合染料硫黄素-T的衍生物，最新的研究表明PiB可以与轻链型淀粉样蛋白特异性结合，用以评估全身淀粉样蛋白的沉积[3]–[4]。本研究通过回顾性分析20例pAL患者的^11^C-PiB PET/MRI结果以及相关临床指标，探究^11^C-PiB PET/MRI在pAL器官受累评估中的价值。

## 病例与方法

1. 病例：对2019年1月至2021年10月就诊于华中科技大学同济医学院附属协和医院并诊断为pAL的20例患者和3名健康志愿者进行回顾性分析，20例患者均至少经过一个器官组织活检证实轻链型淀粉样物质沉积，并排除多发性骨髓瘤、华氏巨球蛋白血症或其他淋巴浆细胞增殖性疾病。pAL诊断标准依据2016年原发性轻链型淀粉样变中国专家诊疗共识和2021年中国系统性轻链型淀粉样变协作组诊疗指南[2]，[5]。临床器官受累评估标准依据2014年英国血液学标准委员会骨髓瘤论坛工作组制定的指南[6]。其器官受累临床标准的阳性定义为：符合国际共识器官受累判断标准或者组织活检证实轻链型淀粉样蛋白沉积。本研究获得华中科技大学同济医学院附属协和医院伦理委员会批准，批件号：[2021]伦审字（0874）号。

2. 临床资料：收集患者性别、年龄、各器官（心脏、肾脏、肺部、肝脏、神经系统、胃肠道、皮肤、舌肌）受累临床表现，心肌肌钙蛋白酶I（cTnI）、N 末端前体脑钠肽（NT-proBNP）、血清游离轻链差值（dFLC）、血清蛋白电泳、血/尿免疫固定电泳、血/尿游离轻链（κ、γ轻链）、24 h尿蛋白、甲状腺功能、肝肾功能、肌电图、肺部CT、心脏超声、心肌磁共振等检查结果。

3. ^11^C-PiB PET/MRI检查方法：所有患者及健康志愿者检查前要求禁食6 h以上。^11^C-PiB由本院PET中心自行制备。采用美国GE公司一体化SIGNA PET/MRI（3.0 T）设备进行扫描。静脉注射显像剂后即行全身PET/MR显像，PET图像行衰减校正及迭代法重建。记录不同器官的最大标准摄取值（SUVmax），高于健康对照相应器官SUVmax值定义为阳性，具体如下：肺>3.0，脾脏>2.5，腺体（甲状腺、唾液腺）>2.5，骨髓>1.4，肌肉和舌>1.0，心脏>0。由于^11^C-PiB主要通过泌尿系统、肝脏、胆囊、肠道等进行生理性排泄[7]–[8]，且脑等器官对其有生理性摄取，因此我们排除了脑、肝脏、胆囊、肾脏、肠道的评估。

4.统计学处理:采用SPSS 25.0及GrdphPad Prism 9进行统计学及数据分析。计量资料以*M*（*Q*_1_, *Q*_3_）表示，计数资料用构成比或率表示。两组间定量资料采用独立样本*t*′检验或非参数秩和检验，多组定量资料采用Kruskal-Wallisa检验。采用Spearman相关分析cTnI、NT-proBNP与SUVmax（心脏）的相关性，采用Pearson相关分析24 h尿蛋白定量与SUVmax（肾脏）的相关性。以*P*<0.05为差异有统计学意义。

## 结果

1. 患者基本资料：20例pAL患者中位年龄59.5（53.5～63.75）岁，男15例（75％），女5例（25％）。初诊患者18例（90％），非初诊患者2例（10％）。20例患者中λ型淀粉样变17例（85％），κ型淀粉样变3例（15％）。所有患者均进行组织活检和刚果红染色，并通过免疫荧光证实轻链的沉积。1例肾、骨髓和肺活检阳性，1例肾和骨髓活检阳性，1例骨髓和舌肌活检阳性，8例肾活检阳性，5例腹壁脂肪活检阳性，3例骨髓活检阳性，1例舌肌活检阳性。18例初诊患者进行分期评估，Mayo2004分期：Ⅰ期6例（33％），Ⅱ期4例（22％），Ⅲa期5例（28％），Ⅲb期3例（17％）。Mayo2012分期：Ⅰ期3例（17％），Ⅱ期7例（39％），Ⅲ期5例（28％），Ⅳ期3例（17％）。患者资料详见表1。

2. ^11^C-PiB在各个器官摄取情况：健康志愿者体内可见示踪剂在泌尿系统、肝胆和胃肠道浓集。另外我们观察到在脑、肺、脾脏、甲状腺、唾液腺、舌肌有弥散轻度浓集，考虑为生理性摄取。对于pAL患者，我们排除了脑、肝脏、胆囊、肾脏、肠道的评估。记录20例患者不同器官的SUVmax，器官受累情况如表1所示。

**表1:** 20例原发性系统性轻链型淀粉样变患者临床基本情况、临床器官受累与^11^C-PiB PET/MRI评估器官受累比较

例号	性别	年龄（岁）	FLC类型	疾病状态	Mayo2004分期	Mayo2012分期	活检阳性部位	临床器官受累与PET/MRI摄取											
								评估	心脏	肾脏	肺部	脾脏	甲状腺	唾液腺	舌肌	肌肉	胃壁	骨髓	神经系统
1	女	51	λ型	初治	Ⅰ期	Ⅰ期	肾脏	临床累及		＋									
								PET/MRI		/									
2	男	60	κ型	初治	Ⅰ期	Ⅰ期	肾脏	临床累及		＋									
								PET/MRI		/									
3	男	58	λ型	非初治	/	/	肾脏、骨髓、肺	临床累及		＋	＋				＋			＋	
								PET/MRI	＋	/	＋	＋		＋	＋	＋		＋	
4	男	55	λ型	初治	Ⅰ期	Ⅱ期	肾脏、骨髓	临床累及		＋								＋	
								PET/MRI	＋	/				＋		＋		＋	
5	男	61	λ型	初治	Ⅱ期	Ⅱ期	肾脏	临床累及	＋	＋									
								PET/MRI		/									
6	男	61	λ型	初治	Ⅰ期	Ⅱ期	肾脏	临床累及		＋									＋
								PET/MRI		/					＋				
7	男	64	λ型	初治	Ⅲb期	Ⅳ期	舌肌	临床累及	＋	＋	＋				＋				
								PET/MRI	＋	/	＋				＋	＋			
8	男	63	λ型	初治	Ⅲa期	Ⅲ期	肾脏	临床累及	＋	＋									
								PET/MRI	＋	/						＋	＋	＋	
9	男	73	κ型	初治	Ⅲb期	Ⅳ期	骨髓	临床累及	＋	＋								＋	
								PET/MRI	＋	/	＋	＋	＋			＋		＋	
10	女	72	λ型	初治	Ⅲa期	Ⅲ期	骨髓	临床累及	＋	＋					＋			＋	
								PET/MRI	＋	/	＋		＋		＋	＋		＋	
11	男	74	λ型	初治	Ⅲa期	Ⅱ期	骨髓	临床累及	＋									＋	
								PET/MRI	＋	/								＋	
12	女	57	κ型	非初治	/	/	皮下脂肪	临床累及	＋		＋							/	
								PET/MRI	＋	/	＋		＋	＋		＋			
13	男	57	λ型	初治	Ⅰ期	Ⅰ期	肾脏	临床累及		＋								/	
								PET/MRI	＋	/									
14	女	50	λ型	初治	Ⅲa期	Ⅲ期	皮下脂肪	临床累及	＋	＋								/	
								PET/MRI	＋	/	＋		＋	＋		＋		＋	
15	男	68	λ型	初治	Ⅱ期	Ⅱ期	皮下脂肪	临床累及	＋									/	
								PET/MRI	＋	/								＋	
16	男	53	λ型	初治	Ⅱ期	Ⅲ期	皮下脂肪	临床累及	＋	＋									
								PET/MRI	＋	/	＋		＋		＋	＋		＋	
17	男	52	λ型	初治	Ⅰ期	Ⅱ期	肾脏	临床累及		＋									
								PET/MRI		/									
18	男	61	λ型	初治	Ⅱ期	Ⅲ期	肾脏	临床累及	＋	＋								/	
								PET/MRI	＋	/	＋								
19	男	59	λ型	初治	Ⅲb期	Ⅳ期	舌肌、骨髓	临床累及	＋	＋					＋	＋		＋	
								PET/MRI	＋	/					＋			＋	
20	女	42	λ型	初治	Ⅲa期	Ⅱ期	皮下脂肪	临床累及	＋	＋			＋						
								PET/MRI	＋	/					＋				

注：FLC：游离轻链；Mayo2004、Mayo2012：系统性轻链型淀粉样变的梅奥预后分期系统；/：未评估或未检查

除肾脏外，^11^C-PiB分别在以下器官摄取阳性：心脏15例（75％），肺部8例（40％），骨髓10例（50％），肌肉10例（50％），舌肌7例（35％），甲状腺6例（30％），唾液腺4例（20％），脾脏2例（10％），胃壁1例（5％）（图1）。所有患者胃腔均有^11^C-PiB分布浓聚，但在例9观察到胃壁有异常显像剂轻度浓聚，其SUVmax为3.5。所有患者均未描述神经系统和皮下脂肪^11^C-PiB摄取。

**图1:**
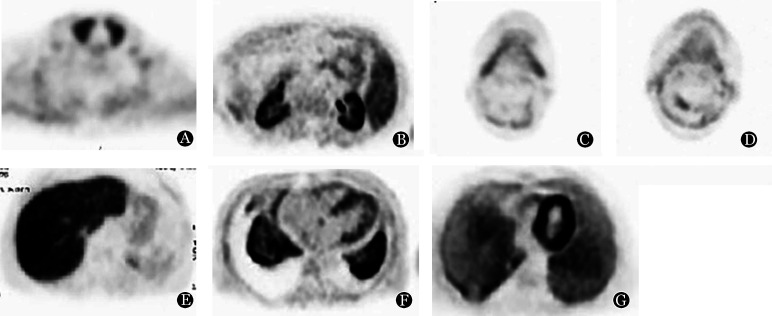
部分原发性系统性轻链型淀粉样变患者具有代表性的^11^C-PiB PET/MRI图像 A：甲状腺显示^11^C-PiB摄取（例9）；B：脾脏、肾脏显示^11^C-PiB摄取（例9）；C：唾液腺显示^11^C-PiB摄取（例3）；D：舌肌显示^11^C-PiB摄取（例3）；E：胃壁显示^11^C-PiB摄取（例8）；F：肺、心脏显示^11^C-PiB摄取（例7）；G：肺、心脏显示^11^C-PiB摄取（例3）

3. 器官受累临床评估与^11^C-PiB PET/MRI评估的比较：我们对20例患者分别按照临床标准和^11^C-PiB PET/MRI标准进行脏器受累的评估，结果如表2所示。从典型的临床表现和实验室检查来看，心脏和肾脏受累的发生率最高，分别为65％和70％；而且^11^C-PiB在心脏和骨髓摄取的阳性率与其临床标准具有很好的相关性。但在肺组织、脾脏、腺体、肌肉和舌肌，^11^C-PiB PET/MRI评估器官受累的阳性率明显高于临床标准。在8例肺实质^11^C-PiB示踪剂异常摄取的患者中，除例3行肺穿刺活检证实淀粉样变，其余患者未进行肺穿刺活检，但例7和例12肺部CT提示双肺间质性病变，排除患者肺部基础疾病，考虑肺实质具有淀粉样变临床器官受累的表现。在7例舌肌^11^C-PiB示踪剂异常摄取的患者中，例7和例19临床表现为舌增大并通过舌肌病理活检证实为pAL；其余5例患者均未进行活检，且仅例3和例10观察到舌增大，余3例患者未观察到舌体肥大等临床器官受累表现。与此相反，通过腹壁脂肪活检证实的轻链沉积患者，均未检出^11^C-PiB摄取阳性。例6双臂出现麻木症状，肌电图提示双侧桡神经感觉传导速度减慢，排除其他可能引起上肢麻木症状的疾病，考虑患者周围神经系统受累；但^11^C-PiB PET/MRI无法评估神经系统的受累情况。表明^11^C-PiB PET/MRI在神经系统和皮肤脂肪评估中具有局限性。

**表2:** 原发性系统性轻链型淀粉样变器官受累的临床标准评估和^11^C-PiB PET/MRI评估情况［例（％）］

受累器官	临床标准	PET/MRI诊断
心脏	13（65）	15(75)
肺	3（15）	8(40)
舌肌	4（20）	7(35)
骨髓	6（30）	10(50)
甲状腺	0（0）	6(30)
唾液腺	0（0）	4(20)
肌肉	0（0）	10(50)
胃壁	0（0）	1(5)
脾脏	0（0）	2(10)
神经系统	1（5）	0(0)
腹壁脂肪	5（25）	0(0)
肾脏	14（70）	/

注：临床标准为符合国际共识器官受累判断标准或者组织活检证实轻链型淀粉样蛋白沉积；/：未评估

从器官受累数目的角度分析，依据^11^C-PiB PET/MRI评估可以比临床标准评估发现更多的器官受累（表2），排除肝脏、肾脏、肠道，PET/MRI评估发现受累器官大于2个的患者有11例（55％），而临床标准评估仅能发现4例（20％）（图2）。

**图2:**
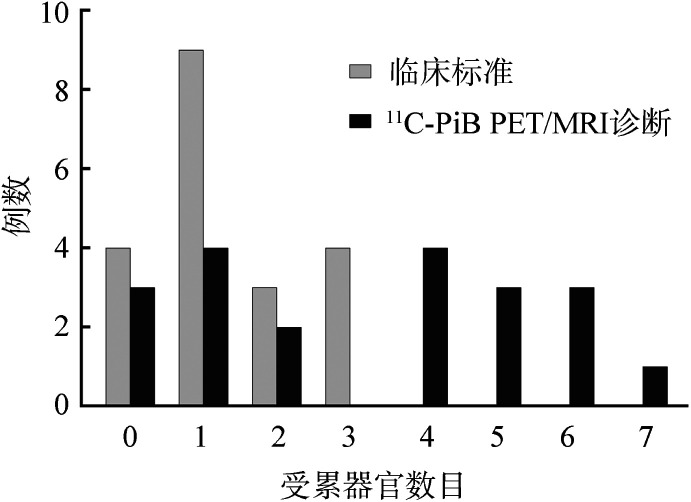
临床标准和^11^C-PiB PET/MRI评估原发性系统性轻链型淀粉样变患者的器官受累数目比较

4. ^11^C-PiB摄取量与心脏受累相关生物学标志之间的关系：为了进一步研究^11^C-PiB摄取量是否能定量评估心脏轻链沉积负荷和心功能情况，我们分析了18例初治患者的疾病分期、心脏生化指标（cTnI、NT-proBNP）、心功能指标（左心室射血分数，LVEF）、心脏形态指标（室间隔厚度，ISV）与心脏SUVmax之间的关系（表3）。结果表明LVEF<50％患者的心脏SUVmax明显高于LVEF≥50％患者（中位数5.0对2.2，*P*<0.05）；ISV≥1.2 cm患者的心脏SUVmax是ISV<1.2 cm患者的4倍（中位数4.0对1.1，*P*<0.05）。另外，心脏SUVmax在不同分期的患者间也具有明显差异，分期越晚，SUVmax值越高（2004分期Ⅰ、Ⅱ、Ⅲa、Ⅲb期中位SUVmax为0、2.35、4.5、4.7，*P*<0.01；2012分期Ⅰ、Ⅱ、Ⅲ、Ⅳ期中位SUVmax为0、1.1、3.8、4.7，*P*<0.01）。进一步相关性分析表明，心脏SUVmax与生化指标cTnI（*r*＝0.83，*P*<0.01）、NT-proBNP（*r*＝0.79，*P*<0.01）具有明显的正相关性。

**表3:** 20例原发性系统性轻链型淀粉样变患者心脏、肾脏和骨髓临床相关指标及PET/MRI相应器官SUVmax情况

例号	NT-proBNP（ng/L）	cTnI（µg/L）	EF（％）	ISV（cm）	心脏磁共振	心脏SUVmax	24 h尿蛋白定量（g）	肾脏SUVmax	骨髓活检	髂骨SUVmax
1	296	0.001	64	1.1	/	0	5.05	7.2	-	1.3
2	76	0.006	67	0.9	/	0	1.54	10.5	-	1.0
3	93	0.004	65	0.9	/	12.0	5.42	6.4	+	1.6
4	31	0.003	65	1.1	未见异常	2.2	4.50	7.6	+	1.9
5	423	0.007	68	/	/	0	5.64	14.2	-	1.1
6	141	0.002	65	0.9	/	0	6.86	8.3	-	0.8
7	10 000	0.138	/	1.3	/	4.6	3.80	13.4	-	1.4
8	414	0.761	70	1.2	/	2.7	2.06	9.0	-	1.5
9	11 300	0.295	41	1.4	/	7.7	1.01	10.7	+	2.4
10	7 640	0.161	33	2.1	/	5.0	5.73	9.3	+	1.5
11	1 180	0.134	69	1.8	/	3.4	0.24	6.6	+	2.0
12	4 070	0.071	40	1.3	左室各壁心内膜下广泛环形延迟强化	1.1	0.38	4.7	/	0.7
13	43	0.002	65	0.9	未见异常	2.2	3.89	12.2	/	0.8
14	3 460	0.720	49	1.1	左室各壁心内膜下广泛环形延迟强化	6.8	1.20	6.6	/	2.3
15	1 730	0.018	66	1.2	左室壁弥漫性延迟强化	2.5	0.43	6.3	/	1.8
16	8 630	0.047	58	/	左室壁弥漫性延迟强化	3.8	1.36	7.4	-	1.7
17	58	0.002	66	0.9	未见异常	0	3.90	9.1	-	0.9
18	2 540	0.066	49	1.2	双房、左室弥漫性延迟强化	2.2	2.18	8.1	/	0.8
19	11 600	0.347	47	1.5	双房、双室弥漫性延迟强化	4.7	1.80	5.8	+	1.6
20	918	0.210	69	0.9	/	4.5	10.33	10.8	-	1.0

注：NT-proBNP：N末端前体脑钠肽；cTnI：心肌肌钙蛋白酶I；EF：射血分数；ISV：室间隔厚度；SUVmax：最大标准摄取值；/：未评估或未检查；-：阴性；+：阳性

值得注意的是，例4 和例13以全身水肿、蛋白尿、低蛋白血症的肾脏受累为主要临床表现，不伴有胸闷、呼吸困难等症状，且反映心脏受累的影像学和生化指标（心脏超声、心肌磁共振、cTnI、NT-proBNP）均未见明显异常，但PET/MRI显示心肌有^11^C-PiB低剂量摄取（SUVmax 2.2），提示^11^C-PiB PET/MRI或可早于临床表现和实验室检查发现心脏淀粉样蛋白的沉积。

例3行PET/MRI前在外院已接受过5次化疗，并获得血液学的非常好的部分缓解。初诊时有心脏受累，此次入院有大量蛋白尿，^11^C-PiB在心肌有明显摄取（SUVmax 12），但心脏超声、NT-proBNP等指标均无异常，表明即使对于治疗后血液缓解的患者，由于体内沉积的淀粉样物质无法完全清除，仍可能出现PET/MRI的阳性摄取。

5. ^11^C-PiB摄取量与骨髓受累相关临床指标之间的关系：共有15例患者进行了髂骨骨髓活检和刚果红染色，其中刚果红染色阳性6例，阴性9例。髂骨的SUVmax值在两组间存在明显差异（中位数1.7对1.2，*P*<0.01）。8例PET评估为阳性（SUVmax>1.4）的患者，其中6例活检刚果红染色阳性；7例PET评估为阴性的患者刚果红染色均为阴性。初步显示骨髓PET评估标准与组织病理学结果有很好的相关性。

6. ^11^C-PiB摄取量与肾脏受累相关临床指标之间的关系:我们进一步分析18例初治患者24 h尿蛋白定量与肾脏SUVmax值的关系（表3）。结果显示肾脏SUVmax与24 h尿蛋白量之间没有明显的相关性（*r*＝0.41，*P*＝0.100）。进一步表明^11^C-PiB的摄取量不能用来定量评估肾脏累及。

## 讨论

近年来，学者们尝试使用全身淀粉样蛋白PET建立一种敏感的、无创的、可视化的评估体系[8]–[9]。目前对于AL有明确优势的示踪剂主要为^18^F标记的florbetapir或florbetaben和^11^C标记的PiB。前者被证实能比目前公认的临床标准更早期发现淀粉样蛋白在全身多种脏器的沉积[9]–[12]。Ezawa等[8]初步证实^11^C-PiB PET/CT成像可用于临床评估AL和转甲状腺素蛋白型淀粉样变（ATTR）患者的淀粉样蛋白分布，但尚不明确能否定量评估受累器官的蛋白沉积或功能损伤。

pAL往往累及多个系统，除了心脏和肾脏受累有较为典型的临床表现和实验室结果外，多数器官如肺、脾脏、腺体等受累都缺乏特异性。本研究首次使用^11^C-PiB PET/MRI评估，结果显示^11^C-PiB PET定性评估器官累及的阳性率明显高于临床标准。在既往研究中，对于脾脏、腺体、舌、肌肉和胃的受累评估也有相似的结论[8]。但对于肺组织受累的评估，Ezawa等[8]发现健康对照者的肺^11^C-PiB摄取也明显增加，认为^11^C-PiB PET无法区分病理性和生理性摄取。而我们的研究中3名健康对照者的肺SUVmax均低于3.0，3例分别经过肺组织活检或者肺CT证实肺组织累及的患者，其肺SUVmax均高于3.0，初步显示肺SUVmax可以用来区分病理性和生理性肺摄取，但由于例数较少，其正常界值有待考量。然而，上述被PET评估为阳性的器官累及，大部分未能得到组织病理学的证实，因此PET/MRI成像体系在评估肺组织、脾脏、腺体、舌、肌肉和胃肠道器官受累方面的优势有待更多的数据加以评估。

心脏是pAL常见的受累器官，受累程度和功能损伤程度与疾病预后密切相关[13]–[14]。由于正常心肌不存在淀粉样物质，没有示踪剂的生理性摄取，所以无论是^18^F标记的示踪剂还是^11^C-PiB在心脏评估中都显示出绝对优势[8]，[15]–[18]。Cuddy等[19]证实^18^F-florbetapir PET/CT的滞留指数可以作为pAL患者心脏轻链淀粉样物质沉积负荷的定量指标；而且对于临床指标评估没有心脏受累的pAL患者，有50％可以通过该技术重新评估为心脏受累。同样，我们的研究中，6例初治临床评估无心脏受累的患者中2例（例4和13）PET发现心肌有^11^C-PiB的摄取；另外，SUVmax值与cTnI、NT-proBNP、LVEF、ISV以及疾病分期都具有相关性，初步显示出^11^C-PiB PET/MRI的SUVmax可以用来早期定性识别心脏的受累，同时定量评估心脏蛋白沉积负荷和功能损伤程度，可能具有判断疾病预后的潜力。

目前AL的诊断金标准依赖受累器官组织活检，但部分组织活检阳性率较低，皮下脂肪为 75％～80％，骨髓仅为 50％～65％[2]。因此如何选择活检部位、提高诊断阳性率是目前有待解决的问题。我们对15例行骨髓活检的患者进行分析显示：髂骨的SUVmax与组织病理学结果有明显的相关性，初步提示依据^11^C-PiB PET对骨髓累及评估有可能提高骨髓活检的阳性率。但是对于腹壁脂肪、舌体的评估价值，因组织活检的病例数太少，无法得出结论。

理想的pAL治疗目标是获得器官缓解，而器官缓解的先决条件是脏器中已沉积的淀粉样物质的清除和轻链毒性的减小[13]，[20]。Cuddy等[19]研究显示在获得血液学完全缓解1年以上的pAL患者心脏中仍有示踪剂的摄取，我们的结果（例3）也证实了淀粉样物质的清除晚于血液学缓解。因此PET/MRI也许可用于对器官残留淀粉样蛋白的定量评估，指导未来pAL治疗药物和治疗终点的选择。

肾脏和肝脏是pAL主要累及器官，但PiB通过肾和肝胆系统进行生理清除，因此^11^C-PiB PET/MRI无法评估尿路和肝胆系统中淀粉样蛋白沉积[8]。同样，^11^C-PiB PET/MRI无法检测外周神经系统中的蛋白沉积，限制了其在评估神经系统受累方面的应用。另外，本研究纳入的样本量较少，缺乏治疗后的长期随访数据，因此对于^11^C-PiB PET/MRI在pAL预后评估、治疗指导方面的价值有待高质量的前瞻性临床研究加以阐明。

综上所述，^11^C-PiB PET/MRI是一种很有前途的评价pAL淀粉样蛋白器官水平沉积的技术。作为一种淀粉样蛋白的可视化系统，器官水平的定性评估可能提高早期无创性诊断pAL的水平；而器官水平（尤其是心脏）的定量评估，有望更精准评估器官功能和预测疾病预后；如能实现动态监测，还可能具有评估治疗疗效和指导治疗方案的潜力。

## References

[1] Merlini G, Comenzo RL, Seldin DC (2014). Immunoglobulin light chain amyloidosis[J]. Expert Rev Hematol.

[2] 中国系统性轻链型淀粉样变性协作组, 国家肾脏疾病临床医学研究中心, 国家血液系统疾病临床医学研究中心 (2021). 系统性轻链型淀粉样变性诊断和治疗指南(2021年修订)[J]. 中华医学杂志.

[3] Antoni G, Lubberink M, Estrada S (2013). In vivo visualization of amyloid deposits in the heart with 11C-PIB and PET[J]. J Nucl Med.

[4] Hellström-Lindahl E, Westermark P, Antoni G (2014). In vitro binding of [³H]PIB to human amyloid deposits of different types[J]. Amyloid.

[5] 中国抗癌协会血液肿瘤专业委员会, 中华医学会血液学分会白血病淋巴瘤学组 (2016). 原发性轻链型淀粉样变的诊断和治疗中国专家共识(2016年版)[J]. 中华血液学杂志.

[6] Gillmore JD, Wechalekar A, Bird J (2015). Guidelines on the diagnosis and investigation of AL amyloidosis[J]. Br J Haematol.

[7] Scheinin NM, Tolvanen TK, Wilson IA (2007). Biodistribution and radiation dosimetry of the amyloid imaging agent 11C-PIB in humans[J]. J Nucl Med.

[8] Ezawa N, Katoh N, Oguchi K (2018). Visualization of multiple organ amyloid involvement in systemic amyloidosis using 11C-PiB PET imaging[J]. Eur J Nucl Med Mol Imaging.

[9] Ehman EC, El-Sady MS, Kijewski MF (2019). Early Detection of Multiorgan Light-Chain Amyloidosis by Whole-Body 18F-Florbetapir PET/CT[J]. J Nucl Med.

[10] Zhao L, Fang Q (2016). Recent advances in the noninvasive strategies of cardiac amyloidosis[J]. Heart Fail Rev.

[11] Baratto L, Park SY, Hatami N (2018). 18F-florbetaben whole-body PET/MRI for evaluation of systemic amyloid deposition[J]. EJNMMI Res.

[12] Wagner T, Page J, Burniston M (2018). Extracardiac 18F-florbetapir imaging in patients with systemic amyloidosis: more than hearts and minds[J]. Eur J Nucl Med Mol Imaging.

[13] Muchtar E, Dispenzieri A, Gertz MA (2021). Treatment of AL Amyloidosis: Mayo Stratification of Myeloma and Risk-Adapted Therapy (mSMART) Consensus Statement 2020 Update[J]. Mayo Clin Proc.

[14] Yilmaz A, Bauersachs J, Bengel F (2021). Diagnosis and treatment of cardiac amyloidosis: position statement of the German Cardiac Society (DGK)[J]. Clin Res Cardiol.

[15] Kim YJ, Ha S, Kim YI (2020). Cardiac amyloidosis imaging with amyloid positron emission tomography: A systematic review and meta-analysis[J]. J Nucl Cardiol.

[16] Manwani R, Page J, Lane T (2018). A pilot study demonstrating cardiac uptake with 18F-florbetapir PET in AL amyloidosis patients with cardiac involvement[J]. Amyloid.

[17] Genovesi D, Vergaro G, Emdin M (2017). PET-CT evaluation of amyloid systemic involvement with [18F]-florbetaben in patient with proved cardiac amyloidosis: a case report[J]. J Nucl Cardiol.

[18] Rosengren S, Skibsted CT, Tolbod L (2020). Diagnostic Accuracy of [11C]PIB Positron Emission Tomography for Detection of Cardiac Amyloidosis[J]. JACC Cardiovasc Imaging.

[19] Cuddy SAM, Bravo PE, Falk RH (2020). Improved Quantification of Cardiac Amyloid Burden in Systemic Light Chain Amyloidosis: Redefining Early Disease?[J]. JACC Cardiovasc Imaging.

[20] Law S, Fontana M, Gillmore JD (2021). Advances in Diagnosis and Treatment of Cardiac and Renal Amyloidosis[J]. Cardiol Clin.

